# Reactivation of cocaine reward memory engages the Akt/GSK3/mTOR signaling pathway and can be disrupted by GSK3 inhibition

**DOI:** 10.1007/s00213-014-3491-8

**Published:** 2014-03-05

**Authors:** Xiangdang Shi, Jonathan S. Miller, Lauren J. Harper, Rachel L. Poole, Thomas J. Gould, Ellen M. Unterwald

**Affiliations:** 1 Department of Pharmacology and the Center for Substance Abuse Research, Temple University School of Medicine, Philadelphia, PA 19140 USA; 2 Department of Psychology, Temple University, Philadelphia, PA USA

**Keywords:** Cocaine, Conditioned place preference, Glycogen synthase kinase-3, Memory, Reconsolidation, mTORC1, Mouse, Reward, Akt, Protein kinase B, Nucleus accumbens, Hippocampus, Fear conditioning

## Abstract

**Rational:**

Memories return to a labile state following their retrieval and must undergo a process of reconsolidation to be maintained. Thus, disruption of cocaine reward memories by interference with reconsolidation may be therapeutically beneficial in the treatment of cocaine addiction.

**Objective:**

The objectives were to elucidate the signaling pathway involved in reconsolidation of cocaine reward memory and to test whether targeting this pathway could disrupt cocaine-associated contextual memory.

**Methods:**

Using a mouse model of conditioned place preference, regulation of the activity of glycogen synthase kinase-3 (GSK3), mammalian target of Rapamycin complex 1 (mTORC1), P70S6K, β-catenin, and the upstream signaling molecule Akt, was studied in cortico-limbic-striatal circuitry after re-exposure to an environment previously paired with cocaine.

**Result:**

Levels of phosporylated Akt-Thr308, GSK3α-Ser21, GSK3β-Ser9, mTORC1, and P70S6K were reduced in the nucleus accumbens and hippocampus 10 min after the reactivation of cocaine cue memories. Levels of pAkt and pGSK3 were also reduced in the prefrontal cortex. Since reduced phosphorylation of GSK3 indicates heightened enzyme activity, the effect of a selective GSK3 inhibitor, SB216763, on reconsolidation was tested. Administration of SB216763 immediately after exposure to an environment previously paired with cocaine abrogated a previously established place preference, suggesting that GSK3 inhibition interfered with reconsolidation of cocaine-associated reward memories.

**Conclusions:**

These findings suggest that the Akt/GSK3/mTORC1 signaling pathway in the nucleus accumbens, hippocampus, and/or prefrontal cortex is critically involved in the reconsolidation of cocaine contextual reward memory. Inhibition of GSK3 activity during memory retrieval can erase an established cocaine place preference.

## Introduction

Compulsive drug use is the hallmark of addiction, and conditioned learning plays a large role in the development of this habitual behavior (Berke and Hyman 2000). Addictive drugs such as cocaine engage molecular signaling pathways that are normally involved in associative learning processes. Exposure to cues previously associated with cocaine availability can lead to a conditioned physiological response accompanied by intense drug craving (Ehrman et al. 1992). Memories for cocaine-associated cues are highly resistant to extinction (Miller and Marshall 2005). Conditioned responses to these cues persist during drug abstinence and contribute to the high rates of relapse to cocaine use even after prolonged periods of abstinence. Thus, a goal of addiction treatment is to extinguish previously learned associations between the positive subjective effects of cocaine and environmental cues signaling cocaine availability.

Memories undergo a reconsolidation process after reactivation and retrieval. Following the reactivation of cocaine-associated memories, exposure to the previous conditioned stimulus (i.e., cue) in the absence of the unconditioned stimulus (i.e., cocaine) reactivates previously learned memories resulting in reconsolidation or strengthening of the memory (Mactutus et al. 1979; Przybyslawski and Sara 1997). During the reactivation process, memory traces are labile and can be manipulated behaviorally or pharmacologically (Nader et al. 2000). As drug-associated cues can trigger relapse to drug-seeking behaviors, pharmacological inhibition of memory reconsolidation processes that maintain intrusive cocaine-related memories may be a useful approach to prevent relapse. Although the neural circuitry of associative learning and cue-induced drug seeking has been investigated, the molecular signaling pathways engaged in this process have not been well-described. As such, the goal of the present study was to investigate the key intracellular signaling proteins involved in the reconsolidation of cocaine-associated memories and to test whether interfering with the signal transduction of these proteins can abolish cocaine-cue memories.

The glycogen synthase kinase 3 (GSK3) pathway has received attention for its role in a variety of neuropsychiatric conditions (Jope and Roh 2006). Two GSK3 isoforms exist in brain, GSK3α and GSK3β. GSK3 is a constitutively active kinase, and its activity is inhibited by phosphorylation of the N-terminal serine-21 of GSK3α and serine-9 of GSK3β (Leroy and Brion 1999; Woodgett 1990). Many substrates of GSK3 are under negative regulation which is released when GSK3 is phosphorylated. GSK3 phosphorylation and hence activity is controlled by several kinases including Akt, also known as protein kinase B, which is a serine/threonine kinase downstream of phosphoinositide 3-kinase (PI3K) (Cross et al. 1995). Although both isoforms of GSK-3 are implicated in neurological and psychiatric disorders, most investigations have focused on the β isoform which is widely expressed throughout the brain. GSK3 has been shown to be a critical molecular substrate involved in psychostimulant-induced behaviors. In our previous studies, inhibition of GSK3 attenuated hyper-locomotion produced by acute administration of cocaine or amphetamine and prevented the development of locomotor sensitization following their repeated administration (Enman and Unterwald 2012; Miller et al. 2009). Likewise, inhibitors of GSK3 reduce methamphetamine-induced locomotor sensitization (Xu et al. 2011). Recent work has shown that administration of a GSK3 inhibitor into the basolateral amygdala immediately after exposure to a cocaine-paired environment disrupts the reconsolidation of cocaine cue memory (Wu et al. 2011). Although the importance of GSK3 has been noted, the signaling pathway involved in the reconsolidation of cocaine-related memories beyond GSK3 has not been investigated.

GSK3β is important for the regulation of an assembly of transcription factors including β-catenin, which is an important component of the Wnt signal transduction pathway (for review, see MacDonald et al. (2009)). GSK3, as an integrator of Akt and Wnt signals, also plays a central role in the regulation of mammalian target of rapamycin (mTOR) during synaptic plasticity (Ma et al. 2011). mTOR is a serine/threonine protein kinase that regulates cell growth and survival by controlling translation in response to nutrients and growth factors (Gingras et al. 2001; Proud 2007). mTOR is a downstream effector of the PI3K/Akt pathway and forms two distinct multiprotein complexes, mTORC1 and mTORC2 (Loewith et al. 2002). mTORC1 includes regulatory-associated protein of mTOR (Raptor) and proline-rich Akt substrate 40 kDa (PRAS40) and promotes protein synthesis and cell growth through phosphorylation of two main substrates, eukaryotic initiation factor 4E-binding protein 1 (4EBP1) and p70 ribosomal S6 kinase 1 (P70S6K). mTORC1 signaling is necessary for memory formation and storage (Parsons et al. 2006; Stoica et al. 2011). In addition, administration of the mTOR inhibitor rapamycin can block the expression of cocaine-induced place preference and locomotor sensitization (Bailey et al. 2011).

In the present study, GSK3 and its major upstream (Akt) and downstream signaling molecules (β-catenin and mTORC1) were measured in the prefrontal cortex, nucleus accumbens, caudate putamen, and hippocampus, in order to determine whether the Akt/GSK3/mTOR and/or Wnt/GSK3/β-catenin signaling pathways are involved in cocaine-associated memory reconsolidation. The importance of GSK3 activity for the maintenance of cocaine-paired cue memories and contextual fear conditioning was also elucidated.

## Materials and methods

### Animals

Male CD-1 mice (8 weeks old) were obtained from Charles River Laboratories (Wilmington, MA). Mice were housed four or five per Plexiglas cage (28 × 18 × 14 cm) without additional enrichment objects in a temperature and relative humidity-controlled room with a 12-h light/dark cycle (lights on at 7:00 am). All animals had access to standard laboratory chow and tap water ad libitum. Animals were housed for 5 days prior to behavioral testing and were handled and weighed daily. Behavioral procedures were conducted between the hours of 9:00 am and 2:00 pm. All animal testing was conducted in accordance with the National Institutes of Health guidelines for the Care and Use of Laboratory Animals and with an approved protocol from Temple University Institutional Animal Care and Use Committee.

### Drugs

Cocaine hydrochloride was generously supplied by the National Institute on Drug Abuse, dissolved in sterile saline (0.9 % NaCl), and injected intraperitoneally (i.p.) in a volume of 3 ml/kg body weight. SB 216763 (Tocris; Ellisville, MO) was dissolved in 3%*v/v* DMSO, 3%*v/v* Tween 80, and distilled water (3:3:94), and injected (i.p.) in a volume of 10 ml/kg body weight. Sterile saline or 3 % DMSO/3 % Tween 80/distilled water were used for control vehicle injections.

### Cocaine conditioned place preference

A randomized unbiased conditioned place preference procedure was used as described by us (Hummel et al. 2006) with some minor modifications. Conditioned place preference chambers were rectangular in shape (45 × 20 × 20 cm) and consisted of two compartments, separated by a removable door. One compartment had a smooth floor with white walls and vertical black stripes, while the other had a rough floor and black walls. On days 1–8, mice were injected with saline or cocaine (10 mg/kg, i.p.) and placed into alternate sides of the conditioning chamber for 30 min. This was repeated once daily for 8 days with mice receiving four pairings with saline and four pairings with cocaine on alternate sides of the conditioning chamber. On test day (day 9), mice were given access to both sides of the conditioning chamber for 30 min in a drug-free state, and time in each side was recorded. Preference scores were determined by subtracting the amount of time spent in the saline-paired compartment from the cocaine-paired compartment.

### Protein measurements by immunoblotting

Brain tissues of interest from individual mice were sonicated in 100 °C 1 % sodium dodecyl sulfate with 1 mM NaF and 1 mM Na3VO4 as phosphatase inhibitors. Samples were boiled for 5 min, aliquotted, and stored at −80 °C. Protein concentrations of tissue samples were determined using a modified Lowry protocol (Lowry et al. 1951). Protein extracts (25–40 µg) were separated on 7.5 % Tris–HCl Bio-Rad Ready-gels (Bio-Rad Laboratories, Hercules, CA, USA) and transferred onto PVDF membranes. Membranes were subsequently blocked for 1 h in Odyssey blocking buffer and Tween–TBS and then incubated overnight at 4 °C in the following antibodies; phospho-Akt (Thr 308) (1:1,000, Cell Signaling, Beverly, MA), phospho-GSK3α/β (1:1,000, Cell Signaling, Beverly, MA), phospho-mTORC1 (1:1,000, Cell Signaling, Beverly, MA), phospho-ß-catenin (1:1,000, Cell Signaling, Beverly, MA), Akt (1:2,000; Cell Signaling, Beverly, MA), GSK3α/β (1:10,000; Santa Cruz, Santa Cruz, CA), mTORC1 (1:1,000, Cell Signaling, Beverly, MA), phospho-P70S6K (1:6,000, Cell Signaling, Beverly, MA), or β-catenin (1:1,000, Cell Signaling, Beverly, MA). Following overnight incubation in primary antibodies, membranes were washed in TTBS and incubated with anti-rabbit or anti-mouse secondary antibodies conjugated to two different infra-red dyes (LI-COR Biosciences, Lincoln, NE, USA) at 25 °C for 1 h in the dark. Secondary antibodies were diluted 1:20,000 in Odyssey blocking buffer with 0.1 % Tween-20 (LI-COR). Membranes were visualized, and proteins were quantified using the Odyssey infrared imaging system and software. Phosphorylated and total forms of the kinases were detected simultaneously as the colors green and red, respectively. Membranes were stripped of antibodies using the New Blot nitro stripping buffer (LI-COR) and re-probed with anti-β-tubulin (1: 400,000; Sigma-Aldrich, St. Louis, MO) to control for potential differences in protein loading and transfer. Ratios of densities of phosphorylated proteins to β-tubulin levels, and total specific proteins to β-tubulin were calculated.

### Contextual fear conditioning

Training and testing of contextual fear conditioning took place in four identical conditioning chambers (17.78 × 19.05 × 38.10 cm) housed in sound attenuating boxes (MED Associates, St Albans, VT), as described in Gould and Higgins (Gould and Higgins 2003). The front, back, and top of the chambers were constructed from Plexiglas panels, and the side walls were composed of stainless steel. The chamber floors, 18 metal rods spaced 0.6 cm apart, were connected to a shock generator and scrambler, and illumination was provided by a 28 V bulb located at the top of the left wall. Ventilation fans (69 dB), providing background noise and air exchange, were located on the right wall of each sound attenuating box. Stimulus administration was controlled by MED-PC software.

The fear conditioning procedure was performed as described previously (Davis et al. 2006). After habituating for 1 h, animals were trained in foreground contextual conditioning.

Training began with a 148-s period (Baseline) that was followed by a 2-s unconditioned stimulus (US) (0.62 mA footshock). Following the first US was another 148-s period that was again followed by a 2-s US (0.62 mA footshock). Thirty seconds following the 2-s US, mice were removed from the training chambers and returned to their home cage. The overall training procedure lasted 5.5 min. The first contextual testing day occurred 24 h after training. Mice were returned to the original training chambers (Context) for 5 min, and freezing behavior was scored. SB 216763 (2.5 or 5 mg/kg, i.p.) or vehicle was administered immediately after contextual testing, and mice were returned to their home cages. Twenty-four hours later, mice underwent a second contextual test wherein freezing was again scored for 5 min after mice were returned to the original training chambers (Context ReTest). Freezing, defined as the complete absence of movement besides respiration, was sampled for 1 s every 10 s during training and testing.

### Experimental design

Experiment 1: The reactivation of cocaine-associated memory. In this experiment, two groups of mice (*N* = 7/group) underwent cocaine conditioned place preference as described above. Twenty-four hours following the test for cocaine place preference on day 9, half of the mice were confined to the previous cocaine-paired compartment in a drug-free state for 10 min to reactivate their cocaine-associated memories (Li et al. 2010; Wu et al. 2011) and were euthanized immediately at the end of the cue exposure. The other half were kept in their home cage and served as a no-reactivation control at the same time. Mice were exposed to CO_2_ for 15 s and decapitated. The prefrontal cortex, nucleus accumbens, and caudate putamen were rapidly dissected on ice from a coronal brain slice, and the hippocampus was obtained by freehand dissection. Brain regions were prepared for measurements of phosphoproteins by immunoblotting as described above.

Experiment 2**:** Effect of the GSK3 inhibitor SB216763 on the reconsolidation of cocaine reward memory. Mice were randomly assigned to six groups (*N* = 7–8/group). All groups of mice underwent cocaine conditioned place preference for 8 days as described previously and were tested for the expression of place preference on day 9. On day 10, four groups of mice were confined to the previous cocaine-paired context for 10 min to reactivate cocaine-associated memory, followed immediately by administration of either vehicle or SB216763 (1, 2.5, or 5 mg/kg, i.p.). The other two groups of mice were injected with either vehicle or SB216763 (2.5 mg/kg, i.p.) in their home cages according to the same time schedule but in the absence of cocaine memory reactivation. On days 11 and 18, all mice were re-tested for cocaine-induced place preference without further drug injections in order to determine if inhibition of SB216763 after memory reactivation could block cocaine place preference.

Experiment 3**:** The effect of SB216763 on the reconsolidation of contextual fear conditioning. The effect of SB216763 on the reconsolidation of fear-associated memories was investigated using contextual fear conditioning as described above, in order to test the specificity of the response to cocaine-associated memories. The study design paralleled the place conditioning procedure in that trained mice were re-exposed to the context, injected with SB216763 immediately following re-exposure, and tested 24 h later for responses to the context. More specifically, mice were trained on contextual fear conditioning procedures and tested for freezing to the context 24 h later. SB216763 (2.5 or 5 mg/kg, i.p.) or vehicle was administered immediately following the test for contextual fear responses, and mice were returned to their home cages. Twenty-four hours later, a second contextual test was performed in the same environment.

### Data analysis

Data were analyzed using a two-tailed Student *t* test, one-way analysis of variance (ANOVA) or two-way ANOVA with exposure, and treatment factors followed by Bonferroni test for multiple comparisons (GraphPad Prism 4, La Jolla, CA), as required by study design. Grubb’s tests were applied to the protein data in order to identify potential outliers, which resulted in the removal of 10 out of 334 data points.

## Results

### Phosphorylation of Akt-Thr308, GSK3α, GSK3β, mTORC1, and P70S6K was downregulated in the nucleus accumbens and hippocampus following reactivation of cocaine-cue memories

**Fig. 1:**
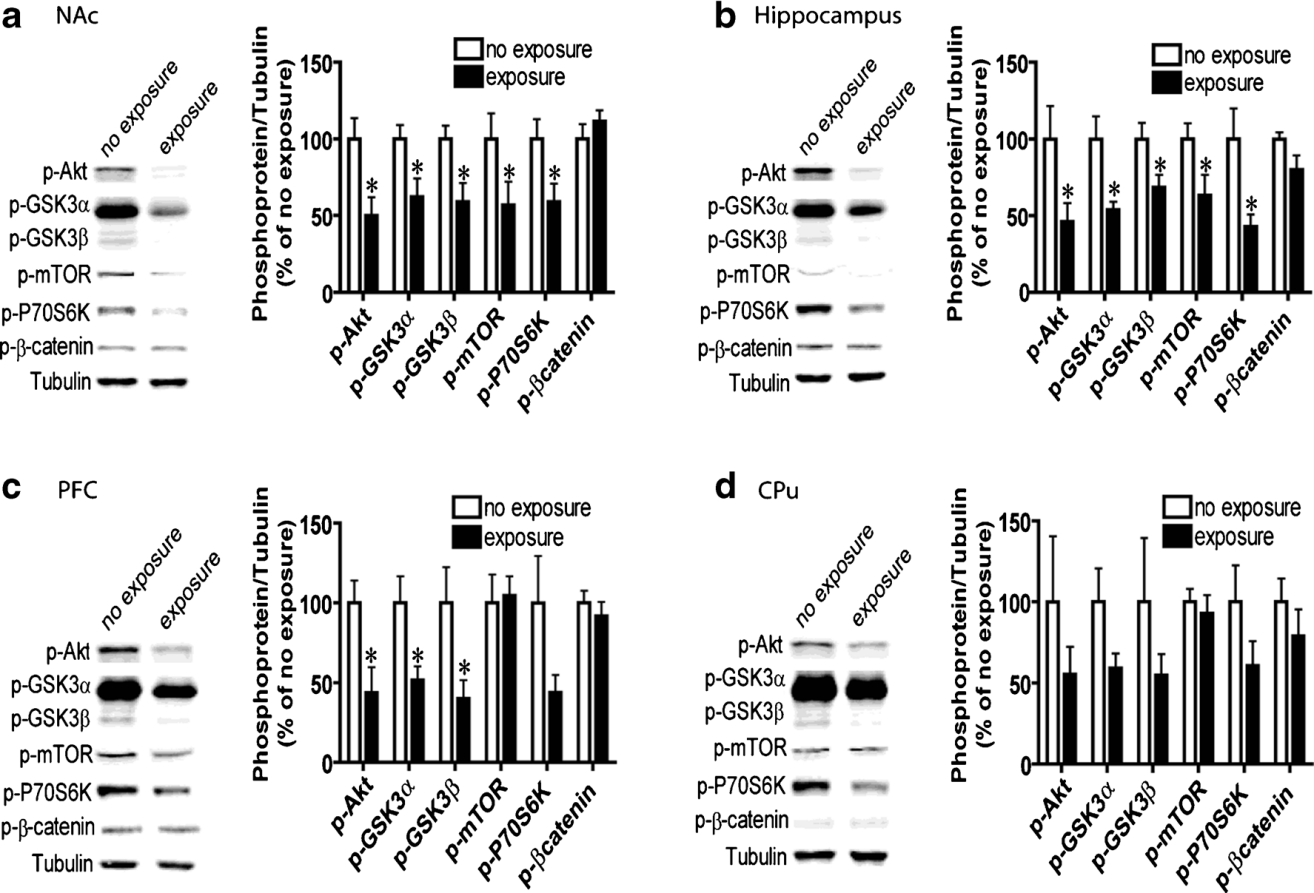
Reactivation of cocaine contextual memory resulted in the dephosphorylation of Akt-Thr308, GSK3α/β, mTORC1, and P70S6K but not β-catenin in a brain region-specific manner. The phosphorylation states of Akt-Thr308, GSK3α/β, mTORC1, P70S6K, and β-catenin were measured in select brain regions following re-exposure of mice to the environment previously paired with cocaine, as compared with non-exposed controls. **a** Levels of p-Akt-Thr308, p-GSK3α, p-GSK3β, p-mTORC1, and p-P70S6K were significantly lower in the nucleus accumbens of exposed versus non-exposed mice (*N* = 6–7/group). *Left*, representative immunoblots of nucleus accumbens tissue from mice with or without exposure to the environment previously paired with cocaine. **b** Representative immunoblots of hippocampus tissue from mice with or without exposure to the environment previously paired with cocaine. Levels of p-Akt-Thr308, p-GSK3α, p-GSK3β, p-mTORC1, and p-P70S6K in the hippocampus were significantly lower in the mice re-exposed to the cocaine context than in non-exposed controls (*N* = 6–7/group). **c** Representative immunoblots of prefrontal cortex tissue from mice exposed or not exposed to the environment previously paired with cocaine. Levels of p-Akt-Thr308, p-GSK3α, and p-GSK3β were significantly reduced following exposure to the cocaine context. No significant differences were found in levels of p-mTORC1, p-P70S6K, or p-β-catenin between the two groups (*n* = 5–7/group). **d** No significant differences were found in levels p-Akt-Thr308, p-GSK3α, p-GSK3β, p-mTORC1, p-P70S6K, or p-β-catenin in the caudate putamen between exposed and non-exposed groups (*n* = 5–7/group). *Bars* represent the mean + SEM of phospho-protein/tubulin integrated density ratios expressed as percent of the ratio in the no exposure control groups. Data were analyzed by unpaired two-tailed *t* test. *p < 0.05, no exposure vs. exposure. *NAc*, nucleus accumbens; *PFC*, prefrontal cortex; *CPu*, caudate putamen

Signaling pathways regulated by reactivation of cocaine-contextual cue memories were identified in specific brain regions in experiment 1. Mice underwent cocaine place preference conditioning for 8 days and were tested for preference on day 9. A significant preference for the cocaine-paired compartment was found (saline- vs. cocaine-paired compartment, 687.3 ± 36.1 vs. 1112.7 ± 36.1 s; *t*(28) = 8.34; *p* < 0.001). On day 10, mice were re-exposed to the context previously paired with cocaine for 10 min or kept in their home cage and brains obtained immediately thereafter. Following re-exposure to the cocaine-paired environment, significant decreases in the phosphorylation of Akt-Thr308 (*t*(11) = 2.70; *p* < 0.05), GSK3α (*t*(12) = 2.50; *p* < 0.05), GSK3β (*t*(12) = 2.74; *p* < 0.05), mTORC1 (*t*(11) = 2.74; *p* < 0.05), and P70S6K (*t*(11) = 2.32; *p* < 0.05) were found in the nucleus accumbens as compared with the levels in mice that underwent cocaine conditioned place preference but were not re-exposed to the cocaine-paired environment (Fig. 1a). Similarly, reduced levels of p-Akt-Thr308 (*t*(11) = 2.27; *p* < 0.05), p-GSK3α (*t*(11) = 2.35; *p* < 0.05), p-GSK3β (*t*(10) = 2.93; *p* < 0.05), p-mTORC1 (*t*(12) = 2.18; *p* < 0.05), and p-P70S6K (*t*(10) = 2.65*;p* < 0.05) were found in the hippocampus following cocaine memory reactivation (Fig. 1b). In the prefrontal cortex (Fig. 1c), exposure to the previous cocaine-conditioned environment lead to reductions in levels of p-Akt-Thr308 (*t*(9) = 2.58; *p* < 0.05), p-GSK3α (*t*(11) = 2.68; *p* < 0.05), and p-GSK3β (*t*(8) = 2.35; *p* < 0.05) but not p-mTORC1 (*t*(12) = 0.8; *p* > 0.05) or p-P70S6K (*t*(8) = 1.61; *p* > 0.05). Although trends towards reductions in p-Akt-Thr308, p-GSK3α, p-GSK3β, and p-P70S6K were seen in the caudate putamen (Fig. 1d), these did not reach statistical significance (all *p*’s > 0.05). No significant differences were found in the levels of phosphorylated β-catenin in any of the brain regions (Fig. 1a–d). The levels of total Akt/tubulin, GSK3α/β/tubulin, mTORC1/tubulin, P70S6K/tubulin, and β-catenin/tubulin did not differ between experimental groups in any brain region (data not shown).

### Inhibition of GSK3 disrupted the reconsolidation of cocaine reward memories

**Fig. 2:**
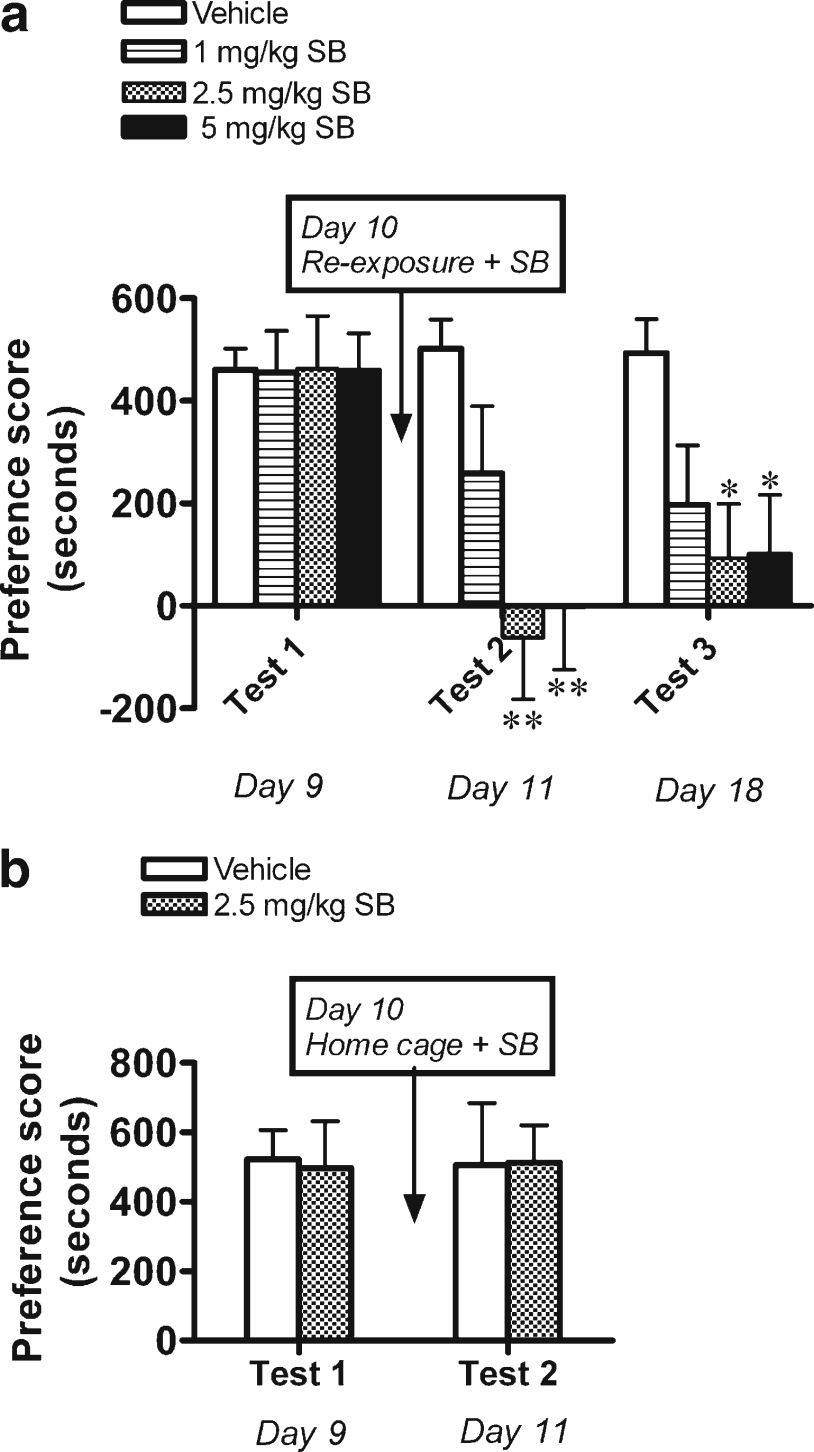
Inhibition of GSK3 immediately following the reactivation of cocaine-associated memory impaired the reconsolidation of cocaine-associated memory. **a** Mice conditioned with cocaine (days 1–8) showed an initial preference toward their cocaine-paired environment (test 1, day 9). On day 10, mice were confined to the environment previously paired with cocaine for 10 min, followed immediately by injection of SB216763 (1, 2.5, or 5 mg/kg, i.p.) or vehicle, and returned to the home cages. Place preference was retested 24 h later (test 2, day 11). Mice injected with 2.5 or 5 mg/kg SB216763 showed no preference for the cocaine-paired environment when retested on day 11 (test 2) or again on day 18 (test 3). Data were analyzed by two-way ANOVA followed by Bonferroni test. *p < 0.05, **p < 0.01 versus vehicle-injected group on the same test day (*N* = 7–8/group). **b** Mice were similarly conditioned with cocaine as above and showed a significant place preference on day 9 (test 1). On day 10, mice were injected with vehicle or SB216763 2.5 mg/kg in the home cages. When retested for place preference on day 11, cocaine place preference was maintained. Data are expressed as means + SEM (*N* = 8/group)

Since GSK3 was found to be activated by re-exposure to an environment previously associated with cocaine, the role of GSK3 in the reconsolidation of cocaine reward memories was investigated using the selective GSK3 inhibitor SB 216763. Following an 8-day cocaine conditioning paradigm, four groups of mice showed similar preferences for the cocaine-paired compartment of the conditioning chamber on day 9 (Fig. 2a). On day 10, all groups of mice were confined to their cocaine-paired compartment in a drug-free state. After 10 min in the cocaine-paired environment, groups of mice were injected with either vehicle or 1, 2.5, or 5 mg/kg SB216763 and immediately returned to the home cage. Twenty-four hours later (day 11), preference was again tested. Two-way ANOVA of preference scores revealed significant main effects of SB 216763 dose (F_3__,__76_ = 6.50, *p* < 0.001) and test day (F_2__,__76_ = 9.60, *p* < 0.001). Post hoc tests revealed that administration of SB 216763 (2.5 and 5 mg/kg) immediately following reactivation of cocaine reward memories significantly attenuated preference for the cocaine-paired compartment when tested 24 h later (*p* < 0.01 vs. vehicle day 11). Cocaine place preference was not significantly altered in mice injected with the lower dose of SB216763 (1 mg/kg) and was maintained in vehicle-injected mice at baseline levels (Fig. 2a, day 11). One week later, preference was retested, and again the vehicle-injected cohort maintained a significant cocaine place preference, whereas mice injected with SB216763 (2.5 and 5 mg/kg) did not *(p* < 0.05 versus vehicle day 18, Fig. 2a). These data indicate that SB216763 can disrupt cocaine reward memories.

Additional groups of mice underwent similar cocaine place conditioning and testing on day 9 (Fig. 2b). On day 10, these mice received the same treatments as the prior study (i.e., vehicle or SB216763 2.5 mg/kg), except the injections were given in the home cage without reexposure to the cocaine-paired environment. When preference was re-tested on day 11, both groups of mice successfully maintained their cocaine place preference (Fig. 2b). These data demonstrate that SB216763 was effective in blocking place preference only when administered after retrieval of cocaine-cue memories, suggesting that the drug is producing its effects specifically by interfering with reconsolidation of cocaine reward memory traces.

### Inhibition of GSK3 failed to impair the reconsolidation of contextual fear memory

**Fig. 3:**
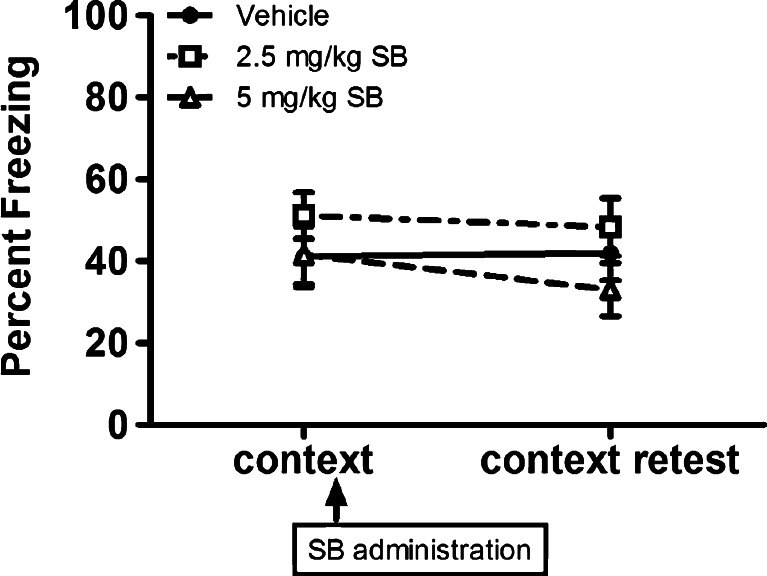
Inhibition of GSK3 with SB216763 did not impair reconsolidation of fear memories. Mice underwent training for contextual fear conditioning. SB 216763, 2.5, or 5 mg/kg, or vehicle was administered immediately after the test for contextual fear conditioning; re-testing occurred 24 h later. No difference in the amount of time spent freezing to the context between vehicle and SB 216763-injected groups was found. Data were analyzed by two-way ANOVA and are expressed as means + SEM of percent time spent freezing during the 5-min test session (*N* = 12/group)

Contextual fear conditioning was used to determine the specificity of the effect of SB216763 on cocaine reward memories. The effects of GSK3 inhibition on reconsolidation of contextual fear memory was investigated by administering SB216763, 2.5, or 5 mg/kg, or vehicle immediately after contextual testing in mice trained in the fear conditioning procedure; freezing to the context was re-tested 24 h after SB216763 administration. A two-way ANOVA revealed that SB216763 had no effect on reconsolidation as assessed by freezing during context re-test (no main effect of SB216763 dose, F_2__,__96_ = 1.748, *p* = 0.18). Thus, SB216763 2.5 or 5 mg/kg administered immediately after contextual testing had no effect on the reconsolidation of fear memories (Fig. 3).

## Discussion

The data presented herein demonstrate a critical role for the GSK3–mTOR signaling pathway in the reconsolidation of cocaine reward memories. GSK3 activity in the nucleus accumbens, hippocampus, and prefrontal cortex was augmented by reactivation of cocaine contextual memories. This was accompanied by reduced phosphorylation of mTORC1, a known target for inhibition by GSK3 (Inoki et al. 2006), and reduced phosphorylated P70S6K in the nucleus accumbens and hippocampus. Thr389-P70S6K is a direct phosphorylation site of mTOR and positively correlates with P70S6K kinase activity (Guertin and Sabatini 2007); phosphorylation of P70S6K is often used as a readout of mTOR activity (Hay and Sonenberg 2004). The importance of this pathway for the maintenance of cocaine-associated contextual memory is highlighted by the demonstration that inhibition of GSK3 with SB 216763 impaired the reconsolidation of cocaine associated memory, thus attenuating the expression of a previously established cocaine place preference 24 h and 7 days later. The ability of SB216763 to disrupt cocaine-associated memory only occurred when the drug was administered at the time of memory reactivation. When administered in the home cage environment, SB216763 had no effect on a previously established cocaine place preference. This provides further support that SB216763 interfered with the reconsolidation process rather than the expression of cocaine place preference.

The disruption of reconsolidation of cocaine reward memory was specific in our study, as the same doses of SB216763 (2.5 and 5 mg/kg) administered immediately after recall of a contextual fear response, failed to impair reconsolidation of contextual fear conditioning, a task that is hippocampus-dependent. This finding suggests that either the association between the footshock and environmental cues is stronger than that for the cocaine-environment trace or that GSK3 activation is not necessary for reconsolidation of fear memories. A previous report demonstrates that heterozygote GSK3β null mice have impaired memory reconsolidation and that another GSK3 inhibitor AR-A014418 impairs contextual fear conditioning in wild-type mice when given prior to memory reactivation (Kimura et al. 2008). The discrepancy between the results of Kimura et al. (2008) and the present study are likely due to the differences in the time of drug administration (1 h before contextual testing vs. immediately after the contextual testing). However, the different outcomes may also be due to differences in the mouse strains (C57BL/6 J vs. CD-1), age (7–10 months vs. 8 weeks), GSK3 inhibitors and/or doses (AR- A014418 vs. SB 216763), and/or procedures (three vs. two training trials).

Accumulating evidence suggests that NMDA receptors play a crucial role in cocaine-related memory reconsolidation (Alaghband and Marshall 2013; Bowers et al. 2007; Itzhak 2008), likely through their bidirectional effects on synaptic plasticity (long-term potentiation, LTP and long-term depression, LTD) (Sajikumar and Frey 2004). In memory reconsolidation, LTD maintains a prior potentiated circuit by competitive synaptic maintenance and protects stable memory traces (Diamond et al. 2005). Previous work has shown that GSK3β regulates the induction of hippocampal NMDA receptor-dependent LTD (Peineau et al. 2007a, b). Stimulation of NMDA receptors reduces Akt activity by decreasing Akt-Thr308 phosphorylation, while activating GSK3β through the dephosphorylation of the Ser9 residue (Peineau et al. 2009). The protein phosphatase 1 (PP1) inhibitor okadaic acid prevents the LTD-associated decreases in both phosphorylation of Akt-Thr308 and GSK3β. Therefore, during LTD, the activation of PP1 could activate GSK3β both by direct dephosphorylation and indirectly through inhibition of Akt (Peineau et al. 2007b). The data presented herein demonstrate that the levels of phosphorylated Akt were reduced, as were phosphorylated GSK3α/β, in the hippocampus, nucleus accumbens, and prefrontal cortex of mice when cocaine contextual memories were reactivated. These results suggest that PI3K-Akt signaling is negatively regulated by the reactivation of cocaine-associated memory. Further experiments are needed to determine whether the dephosphorylation of Akt and GSK3 in our study is dependent on activation of phosphatases such as PP1.

In addition to Akt and GSK3, phosphorylation of mTORC1 was significantly downregulated in the hippocampus and nucleus accumbens following reactivation of cocaine-related memory. mTORC1 has been linked to memory formation and reconsolidation. For example, the mTORC1 inhibitor rapamycin injected into the nucleus accumbens core decreases cue-induced reinstatement of cocaine seeking (Wang et al. 2010). Likewise, rapamycin suppresses the expression but not the development of cocaine-induced place preference (Bailey et al. 2011). In addition, activation of mTORC1 is required for reconsolidation of fear memory, as rapamycin blocks the consolidation and reconsolidation of fear memory (Glover et al. 2010; Li et al. 2013; Parsons et al. 2006). However, this is the first report demonstrating that mTORC1 activity is reduced in the hippocampus and nucleus accumbens during reactivation of cocaine reward memories.

GSK3β together with β-catenin are components of the “destruction complex” which is regulated by canonical Wnt signaling (Logan and Nusse 2004). β-catenin is sequentially targeted for degradation by casein kinase 1α- and GSK3β-mediated phosphorylation. Upon activation of Wnt receptors, the destruction complex dissociates, β-catenin accumulates, and then translocates into the nucleus where it promotes expression of Wnt response genes (Logan and Nusse 2004). As the Wnt/β-catenin signaling pathway is involved in synaptic plasticity (Chen et al. 2006) and consolidation of fear memory (Maguschak and Ressler 2008) and is controlled by GSK3β, its regulation was investigated in the present study. Re-exposure to the environment previously associated with cocaine reward was accompanied by activation of GSK3β. Although GSK3β is able to phosphorylate β-catenin thus marking the protein for degradation, neither changes in the levels of phosphorylated nor total β-catenin was seen following re-exposure to the cocaine-paired environment. Therefore, the Wnt/β-catenin signaling pathway might not be involved in the reactivation or reconsolidation of cocaine-related memory.

Previous work has indicated that the ERK signaling pathway is important for cocaine-associated contextual memory retrieval and/or reconsolidation. Inhibition of ERK activation at the time of re-exposure to an environment previously associated with cocaine attenuates a later preference for that environment (Miller and Marshall 2005; Valjent et al. 2006). It is currently unknown whether there is cross-talk between the ERK and GSK3 cascades in this regard or if they work independently to strengthen reconsolidation, perhaps in different brain areas. Further investigations are needed to resolve the relationship between these two signaling pathways in the context of cocaine reconsolidation.

Retrieval of cocaine cue memory engages a number of brain structures, including the prefrontal cortex, hippocampus, nucleus accumbens, basolateral amygdale, and ventral pallidum (Meyers et al. 2003; Soderman and Unterwald 2008; Weiss et al. 2000). In the present study, changes in Akt/GSK3/mTORC1 signaling pathway occurred in the hippocampus, nucleus accumbens, and prefrontal cortex following exposure to the cocaine-paired environment, suggesting that these regions may play important roles in the process of drug-related memory retrieval and/or reconsolidation. Plasticity of cortical synaptic inputs to dorsal striatum (caudate putamen) is thought to play a role in striatum-dependent learning and memory (Gerdeman et al. 2003; Graybiel 1998), but this kind of learning and memory does not require protein synthesis-dependent reconsolidation upon retrieval (Hernandez and Kelley 2004). Hence, it was not unexpected that the caudate putamen did not show the same regulation of the Akt/GSK3/mTORC1 pathway after exposure to cocaine-paired contextual cues.

**Fig. 4:**
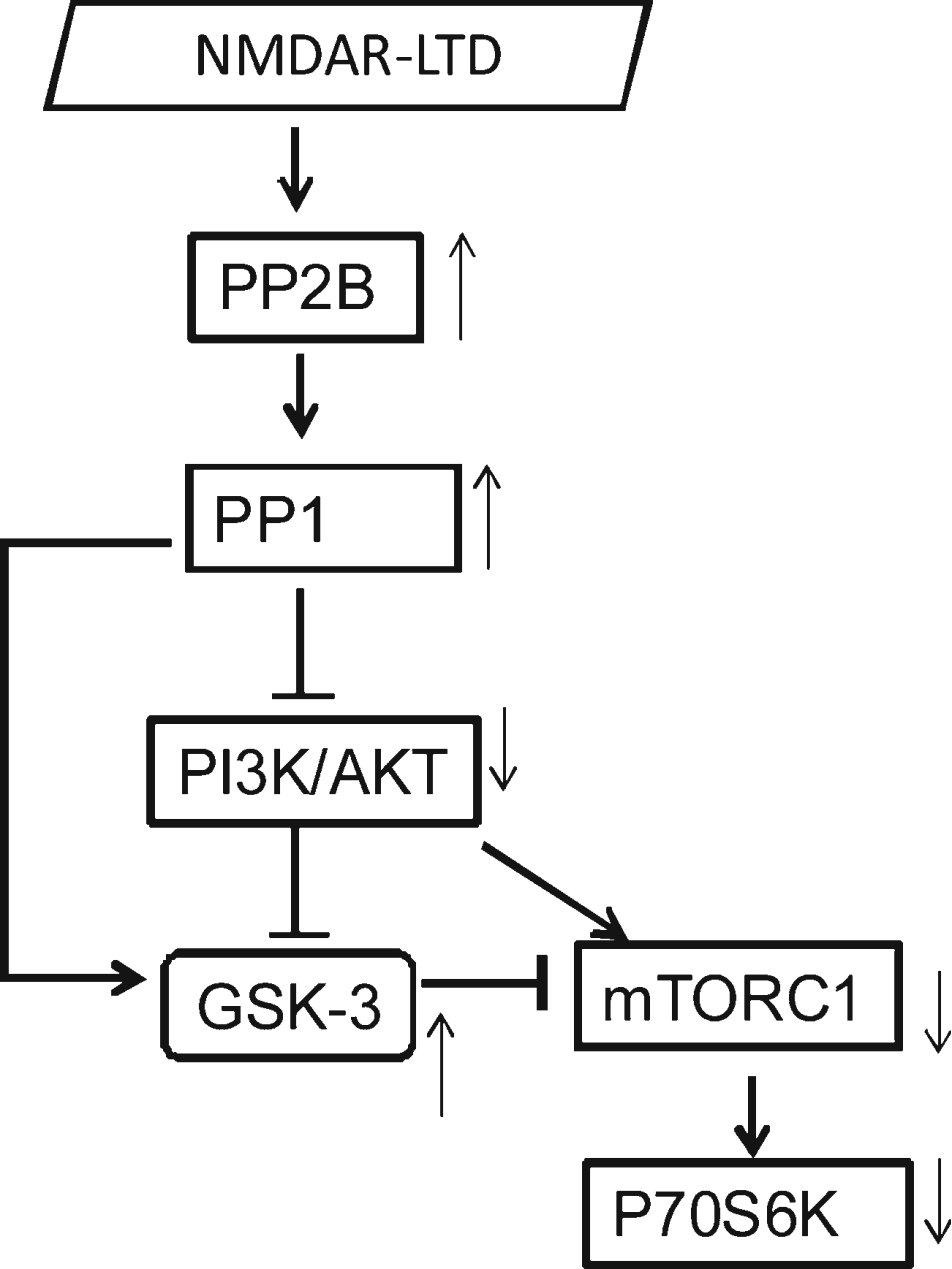
Hypothesized model of molecular signaling underlying the reconsolidation of cocaine-related contextual memory. NMDA receptor-dependent LTD plays an important role in the reconsolidation of cocaine-associated memory. The results presented herein support a model by which a protein phosphatase cascade, such as PP2B and PP1, is activated during LTD and results in the dephosphorylation of Akt and GSK3 following the reactivation of cocaine contextual memories. The activation of GSK3 inhibits the activity of mTORC1. *Arrows* indicate the direction of regulation during reconsolidation. *GSK*, glycogen synthase kinase; *mTORC1*, mammalian target of rapamycin complex 1; *PI3K*, phosphatidylinositol 3-kinase; *PP1*, protein phosphatase 1; *PP2B*, protein phosphatase 2B

The findings presented herein are consistent with the following hypothesized model of the molecular mechanisms underlying the reconsolidation of cocaine-related contextual memory (Fig. 4). Recall of cocaine contextual memories causes the induction of LTD which involves a protein phosphatase cascade. Ca^2+^ entering the cell via NMDA receptors triggers the calcium/calmodulin-sensitive enzyme calcineurin (PP2B). This dephosphorylates inhibitor-1, which leads to activation of PP1. PP1 is an activator of GSK3β via the dephosphorylation of GSK3β-Ser9 (Peineau et al. 2007b). Thus, the dephosphorylation of Akt and GSK3 that occurred upon activation of cocaine-associated reward memory may be initiated by the activation of phosphatases such as PP1 during the induction of NMDA receptor-dependent LTD (reconsolidation of cocaine-related memory). The activation of mTORC1 and P70S6K is reduced accordingly as mTORC1 is a direct substrate of GSK3.

The results presented here demonstrate that Akt/GSK3/mTORC1 signaling pathway in hippocampus, nucleus accumbens, and prefrontal cortex is engaged by reactivation of cocaine reward memories. Inhibition of GSK3 after reactivation of cocaine reward memories interferes with memory reconsolidation and prevents later cocaine-seeking activity. Thus, this pathway is critical for the reconsolidation of cocaine-associated contextual memories. Further study of these signaling pathways and circuitry may provide important insights into the development of effective therapeutics to prevent relapse to cocaine-seeking triggered by environmental cues.
